# Therapeutic vaccines for allergic disease

**DOI:** 10.1038/s41541-017-0014-8

**Published:** 2017-05-08

**Authors:** Danuta Gutowska-Owsiak, Graham S. Ogg

**Affiliations:** MRC Human Immunology Unit, NIHR Biomedical Research Centre, Radcliffe Department of Medicine, Weatherall Institute of Molecular Medicine, University of Oxford, Oxford, UK

## Abstract

Allergic diseases are highly prevalent worldwide and affect all age groups, contributing to a high personal and socioeconomic burden. Treatment with an “allergy vaccine” or allergen immunotherapy aims to provide long-lasting benefits by inducing unresponsiveness to the relevant antigen. The consequences of the therapy are considered disease modifying and range from dampening of the immediate immune responses to the reduction of secondary tissue remodeling. Furthermore, allergen immunotherapy interventions have a potential to slow or cease the development of additional allergic manifestations with a long-term overall effect on morbidity and quality of life. Here, we review proposed mechanisms underlying the therapeutic effects of immunotherapy for allergic diseases. Further, we discuss both standard and novel approaches and possible future directions in the development of allergen immunotherapy.

## Introduction

Allergic diseases, such as atopic dermatitis (AD), allergic asthma (AA), allergic rhinitis (AR), and food allergy (FA) are highly prevalent worldwide. While multiple therapeutic approaches are available to treat the allergy symptoms, the “allergy vaccine” or “allergen-specific immunotherapy” (AIT; colloq. “allergy shot”) is currently the only option offering a disease-modifying intervention. The ultimate goal of the AIT protocol is to provide specific curative therapy with associated long-term tolerance. However, even a partial reduction in disease severity and medication use represent clinically relevant beneficial outcomes of therapy. Moreover, both a reduction in symptoms per se, and slowed progression of the “allergic march” (i.e., appearance of subsequent allergic manifestations in a given patient in time)^1^ as well as a decrease in induction of new sensitizations have a great impact on well-being and quality of life.^1, 2^ Finally, resultant reduction in the use of classically administered medication, such as corticosteroids or antihistamines, leads to a decrease in the associated side effects, risks, and costs. As a consequence, there are already indicators that the AIT leads to a reduction of the public healthcare burden.^3, 4^

The history of AIT started over a century ago, when Leonard Noon reported the first successful attempt to prevent AR by inoculation of an allergic patient with pollen extract before the pollen season.^5^ Throughout those early experiments Noon was able to determine that the sensitivity threshold could be raised by a gradual increase of the dose over time. In 1954 Frankland and Austin^6^ conducted the first double-blinded placebo-controlled trial for treatment of AR and associated asthma, using crude pollen extract (pollaccine) and isolated protein component. Over a decade later, by following a cohort of asthmatic children, Johnstone and Dutton demonstrated the value of desensitisation in decreasing asthma persistence over time.^7^ Finally, a 10-year follow-up, the Preventive Allergy Treatment, study has proven that specific AIT has a potential to slow or cease the allergic march and the development of additional allergic manifestations.^8^

Here, with a focus on immediate IgE-responses, we are reviewing a basic AIT protocol and the mechanisms thought to be responsible for the therapeutic effects of immunotherapy for allergic diseases. Further, we are discussing both long-known and novel approaches and future directions in the development of AIT.

## Mechanism of IgE-mediated allergic reactions

Development of sensitization begins with penetration of antigen through a body barrier which is accompanied by release of epithelial alarmins and influx of early inflammatory cells. This is followed by T cell priming, initiated upon first antigen encounter by antigen presenting cells (APCs) serving this barrier (i.e., a “sensitization phase”). Activation of the immune system during recall responses requires subsequent antigen entry and is promoted by the activated allergen-specific effector memory T cells, antigen-specific IgE antibodies, tissue resident mast cells and basophils. Because of the greater magnitude of the secondary responses and fast time course of the IgE-mediated allergic reaction following the antigen recognition, these immediate consequences can be life-threatening in highly allergic patients.

### Allergen encounter and the epithelial response

Exposure to an allergen through a body barrier (the skin, gut, nasal or respiratory epithelium or oral mucosa) starts a sequence of events critical to the character of the T cell response and additional secondary outcomes. It seems that enzymatic proteolytic activity of allergen components can actively increase the penetration and affect barrier quality by reducing cellular adhesion^9–11^ and induction of potentially barrier-disrupting mediators.^12, 13^ Proteases are also involved in the itch sensation, one of the hallmarks of allergic reactions, independently of histamine via PAR-2 pathway,^14^ therefore perpetuating the itch-scratch cycle and associating with clinical deterioration.

Emerging new evidence suggests that antigen exposure via the skin route in the context of disrupted epidermal barrier particularly predisposes to the induction of allergy. This may be partly explained by the pro-inflammatory role of the Major histocompatibility complex (MHC)-like molecule CD1a, which is highly expressed by Langerhans cells of the epidermis and by subsets of dermal dendritic cells.^15^ Allergen-derived phospholipase, present in insect and snake venoms as well as in other antigen sources such as house dust mite extract, generates antigenic lipids that are presented by CD1a^16^ to T cells further driving subsequent peptide-specific T cell and protein-specific IgE responses. This seems to be especially evident if there is already an underlying dysfunction with the integrity and function of the skin barrier, contained within the epidermis (such as resulting from a mutation in *filaggrin* ^17^ or other “barrier genes”) or if there is inflammation. Combined, the evolving data support the “dual allergen-exposure hypothesis”,^18^ which predicts that the route of primary allergen exposure dictates the clinical outcome. Because of this additional, yet still relatively poorly understood complexity, for the purpose of this review we will specifically focus on the scenario where antigen encounter leads to a productive/activating response, resulting in allergic sensitization.

**Fig. 1:**
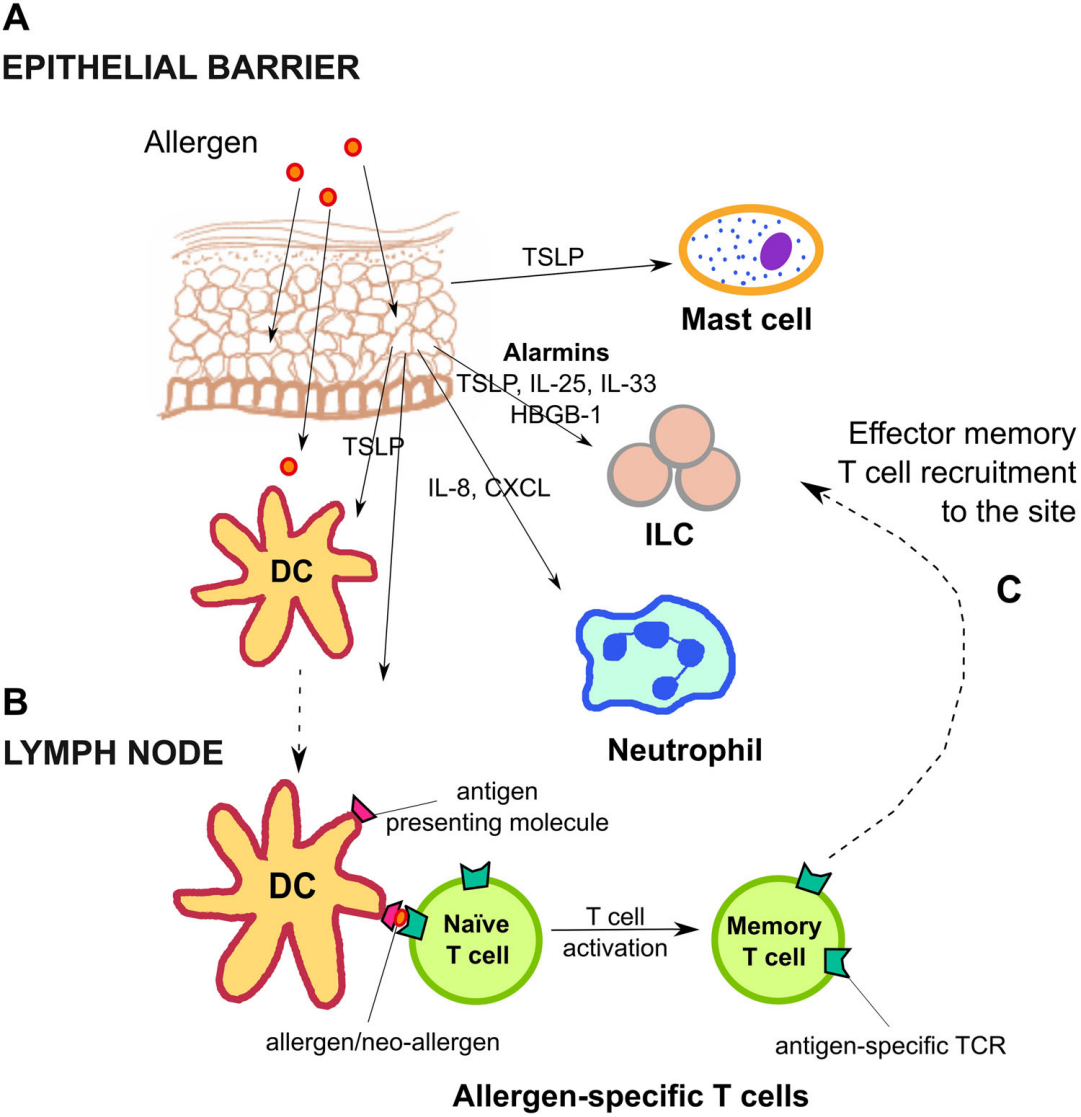
Antigen penetration through epithelial barrier and allergen sensitization

Penetration of an allergen itself or other accompanying components of the allergen extract, such as lipids, initiates a sequence of events in both the immune and non-immune components of the barrier (Fig. 1a). Specifically, activated allergen-exposed epithelia start releasing cytokines and alarmins, i.e., TSLP, IL-25, IL-33,^19^ as well as proinflammatory and chemotactic signals (cytokines: IL-1α, IL-6, IL-8, TNFα; chemokines: CCL-8 and CCL-20, CXCL-1-3).^20, 21^ In addition, stimulated epithelia secrete a potent and multifunctional alarmin, high-mobility group box-1 (HMGB1) protein, which induces proliferation, differentiation, and recruitment of inflammatory cells and forms immunostimulatory complexes; HMGB1 can also enhance cytokine production resulting from TLR engagement.^22^ The protein was shown to be important in the pathogenesis of asthma^23^ and could potentially become a therapeutic target in AD, as demonstrated in a murine model.^24^

Following alarmin release, immune cells accumulate locally, which results in the inflammation of the tissue. Interestingly, epithelial cytokines influence the Th2 predominance of the following response, which ultimately facilitates allergic sensitization. This is partly a result of the response of the tissue resident professional APCs (such as Langerhans cells or subsets of dendritic cell populations) to the epithelium-derived signals, leading to the adaptation of a Th2-promoting phenotype^25^; this has a long-lasting impact on the direction of subsequent immune responses. In addition, innate lymphoid cells type 2 (ILC2s), which are potent secretors of IL-5, IL-4, and IL-13 are resident, and are also further recruited following stimulation by epithelial alarmins. Recently, ILC2s have been shown to be activated when interacting with aberrantly differentiated keratinocytes in vitro, due to a reduction in the KLRG-1-mediated inhibitory signaling they receive in this context^26^ and to keratinocyte overexpression of B7-H6, a ligand of NKp30.^27^ It has also been observed in murine asthma models that NK cells have a capability to prevent ILC recruitment after allergen stimulation.^28^ Development and recruitment of mast cells is also promoted by epithelial TSLP.^29^ In addition, observations following allergen extract application to respiratory epithelium show that neutrophils also accumulate early in the tissues^30^; this early migration of neutrophils involves IL-8 and CXCL chemokines,^31^ which can be induced in epidermal keratinocytes.^21, 32, 33^ These release enzymes such as metalloproteases or elastases and reactive oxygen species^30, 34^ contribute to the tissue damage and remodeling as well as mucin production.^35^ Furthermore, the accumulating granulocytes secrete serine proteases (cathepsin G and elastase), chymase and trypsin which have been shown to generate highly potent isoforms of IL-33^36^ both in humans and murine models.

### Sensitization phase

Following entry, allergens are taken up by the local APCs. Interestingly, besides their immunogenic role, enzymes contained within allergen extracts and venoms may also provide a source of in situ generated new antigens (neo-antigens), which can amplify subsequent adaptive immune responses, as described above. Specifically, phospholipase PLA2 has also been shown to be important source of generated new lipids for CD1a-mediated antigen presentation.^16, 37, 38^ The uptake of the antigens and/or enzymes results in APC activation and their migration to local lymph nodes where these cells display the allergen on their surface, within the groove of antigen presenting molecules. The nature of the presentation (MHC class I, MHC class II, CD1a, CD1b, CD1c, CD1d, or MR1-mediated) is determined by the source and structure of the antigen itself, e.g., NKT cells have been shown to be required for early responses to environmental allergens in animal models.^39, 40^ It is also of note that early NKT cell involvement may stretch beyond specific antigen recognition, since these cells have a capability to suppress protein-induced airway hyperreactivity and skin reactions in mice.^41, 42^ Furthermore, a combined lack of NKT and NK cells in the NK1.1 knock out animals results in inhibition of recruitment of eosinophils and T cells to the lungs as well as reduced Th2 bias and IL-12 production in situ in a model of AA.^43^ Interestingly, NK cells have also been shown to migrate to lymph nodes during the allergen sensitization phase; their role, however, is presently not clear.^44^

Allergen presentation to the naïve T cells in the lymph nodes (Fig. 1b) results in the activation and clonal expansion of the antigen peptide-specific T cells, which recognize epitopes contained within the allergen. It seems that the vast majority of the expanding T cells are potent CD4^+^ cytokine secretors; this leads to profound changes in the local cytokine environment. Specifically, because the APCs were initially “primed” by the Th2-skewing inflammatory milieu, as described above, a Th2 phenotype bias is subsequently induced in the interacting T cells. However, a significant proportion of CD8^+^ allergen-specific T cells have also been shown in allergic patients^45^ and murine models.^46^ Following the antigen presentation and consequent clonal expansion within cytokine-rich domains of the lymph node, activated T cells lose the expression of lymphoid-tissue retaining chemokine receptors (e.g., CCR7) and migrate as effector memory cells to tissue sites (Fig. 1c), recruited by chemoattractants, and amplify tissue-resident T cell responses.^47^ The development of type 2 cytokine-producing allergen peptide-specific T cells promotes class-switching and the acquisition of allergen-specific IgE.

### Secondary phase

A predominant effect following the secondary allergen encounter results from the CD4^+^ effector memory T cell function; these experienced cells of a largely type 2 phenotype actively release large amounts of cytokines (IL-4, IL-5, IL-13, IL-9) during recall responses to their cognate antigen. This is perpetuated by locally abundant and highly potent IL-33 isoforms, generated by neutrophil-derived proteases^36^ and other alarmins, which further recruit and stimulate cells to increase the expression of IL-4 and IL-13. While all these mechanisms lead to the evident type 2 predominance, additional T cell-secreted cytokines (i.e., IFNγ, IL-22, IL-17A) are still relatively enriched and other Th subpopulations (Th1, Th17, Th22, Th9)^48^ have been also implicated during allergic inflammation. Finally, a direct cytotoxic effect, exerted on the epithelial cells, and attributed to CD8^+^ T cells, can be noted as a component of allergic responses. However, IFNγ-dependent regulatory roles have also been proposed for this population in mice.^49^

The specificity of the infiltrating T cells extends far beyond the recognition of peptides, as lipid-derived allergens can be recognized by CD1a-restricted^16, 37, 38^ and CD1d-restricted^40, 50–52^ T cells. There is also a possibility that other unconventional T cell populations,^53^ such as those recognizing MR1 molecule-presented vitamin B derivatives,^54^ i.e., mucosal-associated T cells,^55^ or CD1b-restricted germline-encoded mycolyl-reactive T cells^56^ could enhance or mediate allergen-specific responses in some cases. Whether these populations can contribute to sensitization and/or response to allergens, however, remains to be formally proven.

**Fig. 2:**
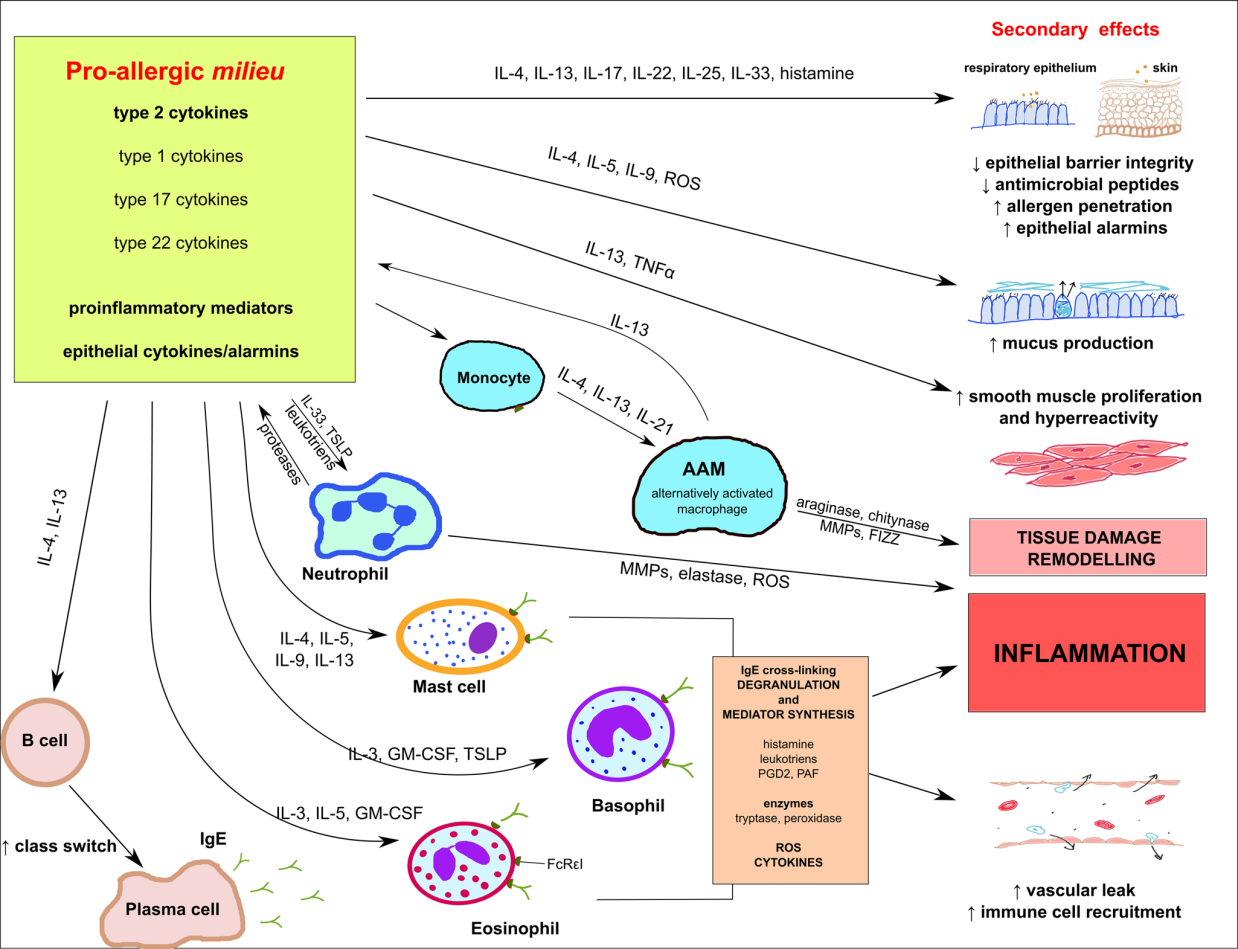
Complexity of allergic milieu and its secondary outcomes

This resulting complex allergic milieu induces several downstream consequences, which ultimately compound the disease symptoms (Fig. 2). Specifically, further recruitment and activation of cell populations acting as effectors during the allergic inflammation (i.e., eosinophils, mast cells, basophils, neutrophils) by IL-3, IL-4, IL-5, GM-CSF, and TSLP is observed.^23, 57–61^ Furthermore, induction of phenotypic and functional changes in monocytes and macrophages result in the generation of alternatively activated macrophages,^62, 63^ which produce arginase-1^64, 65^ and contribute to tissue remodeling, angiogenesis, further Th2 bias of inflammatory responses or local immunosuppression.^66^ It has been also evidenced that the Th2 bias promotes survival of CD8^+^ T cells,^67^ further adding to the increase in the cellular infiltrate.

Importantly, IL-4 and IL-13 induce further IgE class switch recombination in B cells, leading to the changes in the Ig profile in allergic patients, with IL-17A also recently identified as contributing to this effect.^68^ Abundantly secreted IgE antibodies bind to the high-affinity IgE receptor (FcRεI) on resident mast cells, eosinophils, and basophils. When the allergen crosslinks these membrane-bound IgE antibodies, cells rapidly degranulate, releasing inflammatory mediators, pre-synthesized and stored within cytoplasmic granules; activated cells also de novo synthesized mediators shortly after the stimulation.^69^ The mediators include histamine, multiple cytokines, leukotrienes, prostaglandins, and other lipid mediators such as platelet-activating factor.^69, 70^ Their combined action results in the increase of vascular leak and immune cell extravasation, leading to the intensified cell influx into the site of inflammation as well as the appearance of the classical signs of inflammation, i.e., calor, dolor, rubor, tumor (heat, pain, redness, and swelling) in the affected tissue. In addition, release of enzymes,^70^ such as tryptase, serine proteases, and peroxidase or elastase, during the degranulation of effector cells contributes to the tissue damage. Furthermore, IgE also has an assisting role during antigen presentation, as it facilitates uptake of relevant antigen by mechanism involving both the high-affinity (FcεRI),^71^ and low-affinity (FcεRII)^72^ IgE receptors expressed by APCs in the skin (dermal dendritic cells and Langerhans cells) and B cells, respectively. This results in increased production of antigen-specific IgE, IgM, IgG1, and IgG2a,^73, 74^ which promotes the induction of long-term adaptive memory. Finally, IgE antibodies seem to be also involved in the perpetuation of allergic inflammation in animal model of asthma in part through these mechanisms.^75^

There are also many additional effects as a consequence of the Th2 inflammatory milieu, both in situ and in the distant organs, including effects on epithelia in the lungs, gut, and skin. Cytokines and histamine induced during allergic inflammation affect the integrity and function of epithelial barriers, for example, by effects on tight junctions and cellular adhesion, as well as their impact on peptides, proteins, and enzymes critical to the maintenance of barrier integrity and function.^76–89^ The existence of a positive feedback loop between allergic and proinflammatory cytokines and epithelial alarmins which perpetuates the inflammation has also been proposed.^90^ This leads to compounded allergen penetration and sensitization^91^ as well as the increased propensity to infections.^82, 92, 93^ IL-4 and IL-13 stimulate goblet cells within the respiratory and gut epithelia to proliferate and produce mucus,^60, 94, 95^ while IL-13 and TNFα causes smooth muscle proliferation and hyperactivity.^60, 96–98^

## Routes of allergen introduction and clinical AIT procedure

Multiple allergen introduction routes are being utilized either clinically or experimentally (Table 1) with subcutaneous (SCIT) and sublingual (SLIT) AIT being best characterized and most commonly used for treatment. SCIT involves subcutaneous injection of the allergen-containing solution; SLIT is carried out with either a solution (Drop-SLIT) or tablets releasing allergen directly onto the oral mucosa. Furthermore, combination preparations, containing multiple allergens (e.g., grass pollen mix) are available for both SCIT and SLIT. SCIT and SLIT can differ in terms of efficacy, safety, and mechanism.^99^ Specifically, significant differences in the induction of allergen-specific antibodies can be seen, with SCIT inducing more IgG4 and more IgE blocking factor, and SLIT inducing higher transient IgE titers.^100^ The effect on facilitated antigen presentation inhibition also seems to be greater for SCIT, as is difference in basophil activation.^100^ Furthermore, while both antigen delivery routes result in significant IL-10 production, decreased IL-5 production has been observed uniquely in SCIT.^101^ These studies suggest differences in B and T cell responses, but additional mechanisms, such as involvement of mucosa-enriched CD1a-positive Langerhans cells within oral mucosa,^102, 103^ could shape immune responses locally. Similar differences could also potentially be implicated in differential responses to antigen delivery via novel routes, such as epicutaneous and nasal AIT. This difference could also result from specificity of allergen handling by Langerhans cells, which seem to be involved in maintenance of homeostasis in the skin^104^ and being enriched in nasal mucosa under allergen exposure.^105^ This lipid-restricted immune component is missing in the SCIT route, due to the low abundance in cells expressing high levels of CD1a below the dermis; the same will apply to the increasingly promising oral antigen delivery route.^106–108^ Due to the nature of AIT, the treatment is always focused on the confirmed allergens a patient reacts to. The basic AIT protocol involves introduction of that allergen in repeated and often escalating doses in a controlled setting (the “build-up phase”). This protocol may be modified depending on the severity of local or systemic reactions, and is followed by a longer-term “maintenance phase”. The completion of a full AIT schedule often allows for discontinuation of the therapy when satisfactory long-lasting tolerance is reached; however, depending on a patient, this may not be achieved during the immunotherapy course. These patients, however, still often benefit from an increase in the threshold of activation upon the allergen encounter. This state of partial tolerance mirrors a natural pattern of partial desensitization in patients not undergoing AIT and study animals,^109, 110^ yet still increase the threshold of reactivity to an allergen, upon regular exposure to small doses which do not trigger a reaction.^111–114^ Both these examples are reflections of basic mechanisms of allergy, which in itself is dose-dependent, as demonstrated in both human^115–119^ and animal^116, 120, 121^ studies. However, exceeding the threshold can lead to serious consequences, i.e., adverse symptoms, both in those “naturally partially desensitized” patients and patients undergoing AIT. Patients may experience a range of symptoms, from local reactions at the injection site to anaphylaxis. These are more prevalent in the case of SCIT compared with SLIT, but still, life-threatening reactions are relatively rare.

**Table 1:** AIT routes, currently in clinic or investigated experimentally (based on the current data deposited in ClinicalTrials.gov)

Route of allergen introduction	Advancement (experimental/clinical phase)
Subcutaneous SCIT	In clinic
Sublingual SLIT	In clinic
Oral OIT	Phase 3 clinical trials, limited in clinic
Epicutaneous EPIT	Phase 3 clinical trials
Intralymphatic ILIT	Phase 3 clinical trials
Local nasal LNIT	Phase 1/2 clinical trials
Intradermal IDIT	Phase 2
Intragastric IGIT	Animal studies

## Mechanisms underlying AIT

There are many profound changes in allergen-dependent immune responses as a result of AIT, both early and long term. An initial response can be observed, as soon as within the first 24 h from the start of therapy and is thought to be a result of mast cell and basophil desensitization. This effect seems to be mediated via either histamine receptor H2R^122^ or FcεRI internalization and leads to a reduction in number of granules containing inflammatory mediators.^123^ While this may contribute to the immediate reduction of a potential IgE-mediated response to the level below anaphylaxis risk, the long-term AIT efficacy depends on a gradual “education” of the allergen-specific immune response that allows tolerance to the relevant antigen.

Specifically, the continuing benefits observed during the course of the therapy are thought to involve regulatory T cell populations (Treg). This is in line with the essential role of these cells in the prevention of allergic inflammation, evident from the studies of IPEX syndrome. Mutations in the *foxp3* gene, encoding a master transcriptional regulator for the development of Tregs, results in a profound Treg deficiency and dysfunction.^124^ Since this disrupts immune homeostasis, the patients suffer from a multi-organ autoimmune inflammation and have widespread tissue involvement. However, while propensity to autoimmune diseases are most commonly known in these patients, allergic manifestations are also observed, i.e., AD, elevated IgE levels, eosinophilia, as well as severe enteropathy and FA.^125–127^

AIT protocols induce antigen-specific Tregs, which then act to suppress antigen responding effector T cells and result in their state of anergy. Mainly CD4^+^Foxp3^+^ iTregs are induced, but also Foxp3^−^ regulatory iTr1 and iTr35 cells^128, 129^ have been noted. The suppression can be observed at the level of the effector T cell proliferation^130^ and affects both CD4^+^ and CD8^+^ populations.^131^ Tregs exert those functions both directly and via their influence on the APCs.^132^ Specifically, secreted suppressing cytokines (IL-10, TGFβ, and IL-35), which affect responses at multiple levels, provide potent immunosuppression mechanisms in both adaptive and innate immunity.^133, 134^ For example, these cytokines downregulate antigen presenting molecules on APCs, thus affecting their ability to stimulate T cells, halting proliferation and promoting a regulatory phenotype. The effect can be observed at both the level of naïve T cell priming as well as the recall responses.^130^ AIT has been demonstrated to result in Th subset redirection into Th1 cells,^135–139^ induction of anergic Th2 cells,^140^ and preferential deletion of the pathogenic T cell clones.^141, 142^ In addition, Treg-derived IL-10 has the ability to downregulate the expression of proinflammatory cytokines secreted by the APCs.^143^ IL-10 is also thought to contribute to B cell class switching to IgG4 which has an IgE-blocking function. Acting directly in a contact-dependent fashion, Tregs also modulate function of these cells by engaging CD80/CD86 and providing inhibitory CTLA-4-mediated signals. Tregs can also compete with pathological effector T cells physically (simply by blocking their access to the DCs),^144^ reducing available IL-2^145, 146^ and stimulating tryptophan degradation by dendritic cell IDO (indoleamine-pyrrole 2,3-dioxygenase),^147–149^ which leads to metabolic disruption. Tregs facilitate cAMP-mediated effector T cell inhibition,^146, 150, 151^ and can engage in adenosine-receptor immunosuppression^152, 153^ and contribute to the direct killing of antigen-specific effector T cells in a granzyme B and perforin-mediated fashion.^154^ Tregs have also been shown to prevent recruitment of mast cell progenitors in a murine model of asthma.^155^ In addition, induction of Tregs may promote beneficial allergy-alleviating changes in further cell populations; it has been previously shown that Tregs have a direct suppressive effect on monocytes/macrophages,^156–158^ mast cells,^159, 160^ and eosinophils.^161^

The second regulatory cell type, which appears to have a beneficial role during AIT is a population of much less studied regulatory B cells (Breg).^162, 163^ These cells similarly secrete IL-10, TGFβ, and IL-35, therefore being Treg counterparts homologous in their ability to affect immune responses. It seems that the IL-10-producing Breg subtype (Br1) increases during AIT.^164^ Allergen-specific Breg immunosuppressive capacity during AIT has been attributed to the production of IgG4 by these cells and the suppression of allergen-specific effector T cells,^165^ as well as the induction of Tregs by promoting conversion of CD4^+^CD25^−^ T cells into CD4^+^CD25^+^ Treg cells.^162^

**Fig. 3:**
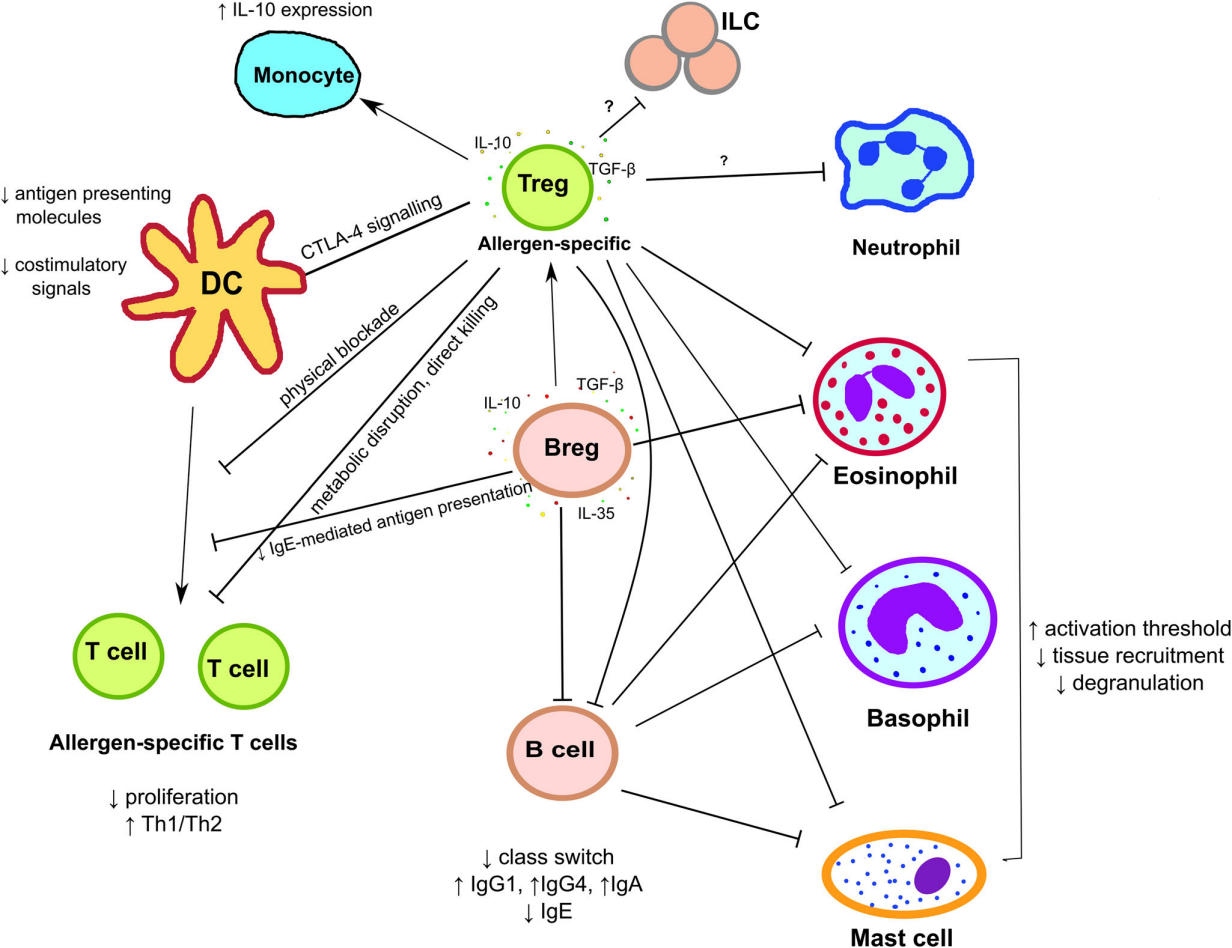
Role of regulatory T and B cell during AIT

AIT leads to a complete or partial reversal of the consequences driven by effector T cell activation (Fig. 3); qualitative and quantitative changes in the inflammatory milieu result in beneficial secondary outcomes. Specifically, a decrease in class switching by B cells and a reduction of IgE production with an evident increase in IgA, IgG4, and IgG1 levels can be observed^130, 131^; these antibodies compete for allergen therefore increasing the threshold required for mast cell and basophil degranulation, IgE-mediated antigen uptake and development of memory IgE production.^166^ An increase in the blocking IgG antibodies can be assessed by a traditional functional test with patient serum (referred to as a “patient self-test” or “P-S test”) and other means.^166–169^ This is critical, as these latter antibodies compete with FcRεI-immobilized IgE antibodies for allergen binding. Specifically, stabilization of the cells capable of degranulation by reducing the chance of IgE cross-linking and increasing activation threshold reduces risk of type I hypersensitivity reactions and IgE-mediated antigen presentation by the APCs. AIT also leads to the alleviation of inflammation by reduced local accumulation of basophils and eosinophils,^170^ with a similar effect on the reactivity and recruitment of neutrophils having also been demonstrated.^171, 172^ These result in a reduction in inflammation in the affected tissue, as shown both in double-blinded patient studies and animal models.^173, 174^ Finally, AIT has also been shown to prevent ILC2 increase during pollen seasons in patients with AR^175^ by as yet unknown mechanisms.

## Novel approaches and future directions

While a tremendous progress has been seen in the AIT field over the last decades, there is still a need for methods that increase convenience and patient safety as well as a spectrum of available AIT allergens. Novel approaches include the identification of new allergen delivery routes and novel antigen preparations as well as combination protocols, where AIT is carried out parallel to other treatments. Currently investigated novel AIT routes include oral (OIT),^176^ epicutaneous (EPIT),^177, 178^ local nasal (LNIT), intralymphatic (ILIT),^177, 179, 180^ and intradermal (IDIT). To date, the intragastric (IGIT) route has only been tested in mice.^181^

While native allergen source^107^ or crude allergen extracts^182, 183^ have been used in AIT protocols, these natural products may vary greatly in terms of allergen content. Recombinant allergens have an advantage of a standardized and well-defined manufacturing process, leading to product consistency and easy scalable production. However, individual antigens may not fully modulate the clinical response to the whole allergen source and side effects can be observed at a level similar to crude extracts.^184^ Furthermore, recombinant allergens pose a risk of inducing IgE production, which limits their use.^185^ Therefore, great effort is being made currently to improve allergen sources for AIT. Approaches to formulate these new allergen preparations focus on the use of specific proteins or allergen extract fractions or modified/engineered allergens, aiming to reduce IgE reactivity while retaining efficacy; these are investigated predominantly in classical SLIT/SCIT studies. Methods include the use of synthetic overlapping peptide epitopes,^186–188^ fusion proteins,^189^ or allergens that have been chemically modified, e.g., denatured (also known as “allergoids”).^190, 191^ Also, modified or fragmented recombinant allergens, hypoallergenic recombinant allergen derivatives,^192^ and allergen-derived peptides are being investigated currently.^190, 193^ While all these T-cell directed approaches are protein/peptide-based, it is also conceivable that we will observe new AIT approaches, constructed around targeting of lipid-specific innate and adaptive immune responses in the future.

Furthermore, innovative B-cell-focused approaches, aiming to induce responses promoting the generation of the IgG antibodies to compete with IgE for allergen binding are also being studied.^194^ In addition, AIT preparations of allergens are being combined with a choice of specific carriers or adjuvants, for example, Toll-like receptor ligands or virus particles in combination with allergen preparations, to redirect immune response and induce a more favorable Th bias.^193^

Finally, introducing combinations of drugs and AIT, e.g., parallel use of AIT and treatment with additional biologics (e.g., anti-IgE antibody)^195^ or even defined bacterial strains in order to modulate the impact of the accompanying microbiome,^196^ is also a potential new approach to develop safe and efficacious immunotherapy protocols.

## Summary

Multiple mechanisms are involved in the induction of antigen sensitization and subsequent allergic reactivity, both from the side of the immune system and the epithelial component of body barriers, which are the sites of allergen entry. AIT currently provides the only cause-directed treatment option for allergy sufferers, and aims to induce peripheral tolerance to the relevant antigen. As such, AIT has been shown to be efficacious and to have a direct effect on patient welfare and socioeconomic burden.

While AIT targets multiple allergic pathways, both the short-term and long-term beneficial outcomes involve the induction of allergen-specific regulatory T and B cells, directly and indirectly suppressing innate and adaptive effector populations. Consequently, additional beneficial outcomes result from the therapy, due to a reduction in chronic inflammation and tissue remodeling, inhibition of new sensitizations and slower progression of the “allergic march”. Novel AIT approaches include allergen modifications, altered formulations, and optimization of the vaccine introduction route to achieve tolerance induction and associated clinical benefit.

## References

[1] Kristiansen, M. et al. Allergen immunotherapy for the prevention of allergy: a systematic review and meta-analysis. *Pediatr. Allergy Immunol*. **28**, 18–29 (2017). 10.1111/pai.12661 27653623

[2] Elenius V, Jartti T (2016). Vaccines: could asthma in young children be a preventable disease?‬‬‬‬‬‬‬. Pediatr. Allergy Immunol..

[3] Hankin CS, Cox L, Bronstone A, Wang Z (2013). Allergy immunotherapy: reduced health care costs in adults and children with allergic rhinitis. J. Allergy Clin. Immunol..

[4] Hankin CS (2010). Allergen immunotherapy and health care cost benefits for children with allergic rhinitis: a large-scale, retrospective, matched cohort study. Ann. Allergy Asthma Immunol..

[5] Noon L (1911). Prophylactic inoculation against hay fever. Lancet.

[6] Frankland AW, Augustin R (1954). Prophylaxis of summer hay-fever and asthma: a controlled trial comparing crude grass-pollen extracts with the isolated main protein component. Lancet.

[7] Johnstone DE, Dutton A (1968). The value of hyposensitization therapy for bronchial asthma in children—a 14-year study. Pediatrics.

[8] Jacobsen L (2007). Specific immunotherapy has long-term preventive effect of seasonal and perennial asthma: 10-year follow-up on the PAT study. Allergy.

[9] Wan H (1999). Der p 1 facilitates transepithelial allergen delivery by disruption of tight junctions. J. Clin. Invest..

[10] Wan H (2000). Quantitative structural and biochemical analyses of tight junction dynamics following exposure of epithelial cells to house dust mite allergen Der p 1. Clin. Exp. Allergy.

[11] Wan H (2001). The transmembrane protein occludin of epithelial tight junctions is a functional target for serine peptidases from faecal pellets of Dermatophagoides pteronyssinus. Clin. Exp. Allergy.

[12] Gutowska-Owsiak D (2014). The histamine-synthesizing enzyme histidine decarboxylase is upregulated by keratinocytes in atopic skin. Br. J. Dermatol..

[13] Kim, J. H. et al. Thymic stromal lymphopoietin downregulates filaggrin expression by signal transducer and activator of transcription 3 (STAT3) and extracellular signal-regulated kinase (ERK) phosphorylation in keratinocytes. *J. Allergy Clin. Immunol*. **136**, 205–208.e209 (2015) 10.1016/j.jaci.2015.04.026 26026341

[14] Kempkes, C., Buddenkotte, J., Cevikbas, F., Buhl, T. & Steinhoff, M. in *Itch: Mechanisms and Treatment* (eds Carstens, E. & Akiyama, T.) (CRC Press, 2014). 24830021

[15] Boltjes, A. & Van Wijk, F. Human dendritic cell functional specialization in steady-state and inflammation. *Front. Immunol*. **5**, 131 (2014). 10.3389/fimmu.2014.00131 PMC3978316 24744755

[16] Jarrett R (2016). Filaggrin inhibits generation of CD1a neolipid antigens by house dust mite-derived phospholipase. Sci. Transl. Med..

[17] McAleer MA, Irvine AD (2013). The multifunctional role of filaggrin in allergic skin disease. J. Allergy Clin. Immunol..

[18] Lack G (2008). Epidemiologic risks for food allergy. J. Allergy Clin. Immunol..

[19] Divekar R, Kita H (2015). Recent advances in epithelium-derived cytokines (IL-33, IL-25 and TSLP) and allergic inflammation. Curr. Opin. Allergy Clin. Immunol..

[20] Vroling AB, Duinsbergen D, Fokkens WJ, van Drunen CM (2007). Allergen induced gene expression of airway epithelial cells shows a possible role for TNF-alpha. Allergy.

[21] Gutowska-Owsiak D, Ogg GS (2012). The epidermis as an adjuvant. J. Invest. Dermatol..

[22] Harris HE, Andersson U, Pisetsky DS (2012). HMGB1: a multifunctional alarmin driving autoimmune and inflammatory disease. Nat. Rev. Rheumatol..

[23] Shim EJ (2012). The role of high-mobility group box-1 (HMGB1) in the pathogenesis of asthma. Clin. Exp. Allergy.

[24] Karuppagounder V (2014). Resveratrol attenuates HMGB1 signaling and inflammation in house dust mite-induced atopic dermatitis in mice. Int. Immunopharmacol..

[25] Soumelis V (2002). Human epithelial cells trigger dendritic cell mediated allergic inflammation by producing TSLP. Nat. Immunol..

[26] Salimi M (2013). A role for IL-25 and IL-33-driven type-2 innate lymphoid cells in atopic dermatitis. J. Exp. Med..

[27] Salimi, M. et al. Group 2 innate lymphoid cells express functional NKp30 receptor inducing type 2 cytokine production. *J. Immunol*. **196**, 45–54 (2016). 10.4049/jimmunol.1501102 PMC4913864 26582946

[28] Simons, B. et al. PGI2 controls pulmonary NK cells that prevent airway sensitization to house dust mite allergen. *J. Immunol*. **198**, 461–471 (2017). 10.4049/jimmunol.1600275 PMC5173397 27895167

[29] Han NR (2014). TSLP induces mast cell development and aggravates allergic reactions through the activation of MDM2 and STAT6. J. Invest. Dermatol..

[30] Hosoki K, Itazawa T, Boldogh I, Sur S (2016). Neutrophil recruitment by allergens contribute to allergic sensitization and allergic inflammation. Curr. Opin. Allergy Clin. Immunol..

[31] Ferreira MA (2003). Cytokine expression in allergic inflammation: systematic review of in vivo challenge studies. Mediators Inflamm..

[32] Barker JN (1991). Modulation of keratinocyte-derived interleukin-8 which is chemotactic for neutrophils and T lymphocytes. Am. J. Pathol..

[33] Kato, T., Takai, T., Mitsuishi, K., Okumura, K. & Ogawa, H. Cystatin A inhibits IL-8 production by keratinocytes stimulated with Der p 1 and Der f 1: biochemical skin barrier against mite cysteine proteases. *J. Allergy Clin. Immunol*. **116**, 169–176 (2005). 10.1016/j.jaci.2005.03.044 15990791

[34] Ventura I (2014). Neutrophils from allergic asthmatic patients produce and release metalloproteinase-9 upon direct exposure to allergens. Allergy.

[35] Voynow JA (1999). Neutrophil elastase increases MUC5AC mRNA and protein expression in respiratory epithelial cells. Am. J. Physiol..

[36] Lefrancais E (2012). IL-33 is processed into mature bioactive forms by neutrophil elastase and cathepsin G. Proc. Natl. Acad. Sci. U. S. A..

[37] Bourgeois EA (2015). Bee venom processes human skin lipids for presentation by CD1a. J. Exp. Med..

[38] Subramaniam S (2016). Elevated and cross-responsive CD1a-reactive T cells in bee and wasp venom allergic individuals. Eur. J. Immunol..

[39] Reyes NJ, Mayhew E, Chen PW, Niederkorn JY (2010). NKT cells are necessary for maximal expression of allergic conjunctivitis1. Int. Immunol..

[40] Wingender G (2011). Invariant NKT cells are required for airway inflammation induced by environmental antigens. J. Exp. Med..

[41] Akbari O (2003). Essential role of NKT cells producing IL-4 and IL-13 in the development of allergen-induced airway hyperreactivity. Nat. Med..

[42] Schouten B (2012). Invariant natural killer T cells contribute to the allergic response in cow’s milk protein-sensitized mice. Int. Arch. Allergy Immunol..

[43] Korsgren M (1999). Natural killer cells determine development of allergen-induced eosinophilic airway inflammation in mice. J. Exp. Med..

[44] Ple C (2010). Natural killer cells accumulate in lung-draining lymph nodes and regulate airway eosinophilia in a murine model of asthma. Scand. J. Immunol..

[45] Seneviratne SL (2002). Allergen-specific CD8(+) T cells and atopic disease. J. Clin. Invest..

[46] Harris SJ (1997). Prediction of murine MHC class I epitopes in a major house dust mite allergen and induction of T1-type CD8+T cell responses. Int. Immunol..

[47] Lian J, Luster AD (2015). Chemokine-guided cell positioning in the lymph node orchestrates the generation of adaptive immune responses. Curr. Opin. Cell Biol..

[48] Jutel M, Akdis CA (2011). T-cell subset regulation in atopy. Curr. Allergy Asthma Rep..

[49] Tang Y (2012). Antigen-specific effector CD8 T cells regulate allergic responses via IFN-gamma and dendritic cell function. J. Allergy Clin. Immunol..

[50] Russano AM (2006). Recognition of pollen-derived phosphatidyl-ethanolamine by human CD1d-restricted gamma delta T cells. J. Allergy Clin. Immunol..

[51] Jyonouchi S (2011). Invariant natural killer T cells from food allergic versus non-allergic children exhibit differential responsiveness to milk-derived sphingomyelin. J. Allergy Clin. Immunol..

[52] Agea E (2005). Human CD1-restricted T cell recognition of lipids from pollens. J. Exp. Med..

[53] Mondot S, Boudinot P, Lantz O (2016). MAIT, MR1, microbes and riboflavin: a paradigm for the co-evolution of invariant TCRs and restricting MHCI-like molecules?. Immunogenetics.

[54] Kjer-Nielsen L (2012). MR1 presents microbial vitamin B metabolites to MAIT cells. Nature.

[55] Treiner E (2003). Selection of evolutionarily conserved mucosal-associated invariant T cells by MR1. Nature.

[56] Van Rhijn I (2013). A conserved human T cell population targets mycobacterial antigens presented by CD1b. Nat. Immunol..

[57] Burton OT (2013). Direct effects of IL-4 on mast cells drive their intestinal expansion and increase susceptibility to anaphylaxis in a murine model of food allergy. Mucosal Immunol..

[58] Nakamura Y (1993). Factors that stimulate the proliferation and survival of eosinophils in eosinophilic pleural effusion: relationship to granulocyte/macrophage colony-stimulating factor, interleukin-5, and interleukin-3. Am. J. Respir. Cell Mol. Biol..

[59] Kimura M, Tsuruta S, Yoshida T (1998). Correlation of house dust mite-specific lymphocyte proliferation with IL-5 production, eosinophilia, and the severity of symptoms in infants with atopic dermatitis. J. Allergy Clin. Immunol..

[60] Yang M (2001). Interleukin-13 mediates airways hyperreactivity through the IL-4 receptor-alpha chain and STAT-6 independently of IL-5 and eotaxin. Am. J. Respir. Cell Mol. Biol..

[61] Voehringer D (2012). Basophil modulation by cytokine instruction. Eur. J. Immunol..

[62] Gordon S, Martinez FO (2010). Alternative activation of macrophages: mechanism and functions. Immunity.

[63] Anthony RM (2006). Memory T(H)2 cells induce alternatively activated macrophages to mediate protection against nematode parasites. Nat. Med..

[64] Arango Duque, G. & Descoteaux, A. Macrophage cytokines: involvement in immunity and infectious diseases. *Front. Immunol*. **5**, 491 (2014). 10.3389/fimmu.2014.00491 PMC4188125 25339958

[65] Joerink M, Savelkoul HF, Wiegertjes GF (2006). Evolutionary conservation of alternative activation of macrophages: structural and functional characterization of arginase 1 and 2 in carp (Cyprinus carpio L.). Mol. Immunol..

[66] Biswas SK, Mantovani A (2010). Macrophage plasticity and interaction with lymphocyte subsets: cancer as a paradigm. Nat. Immunol..

[67] Huang L-R, Chen F-L, Chen Y-T, Lin Y-M, Kung JT (2000). Potent induction of long-term CD8(+) T cell memory by short-term IL-4 exposure during T cell receptor stimulation. Proc. Natl. Acad. Sci. U. S. A..

[68] Milovanovic M, Drozdenko G, Weise C, Babina M, Worm M (2010). Interleukin-17A promotes IgE production in human B cells. J. Invest. Dermatol..

[69] Moon TC, Befus AD, Kulka M (2014). Mast cell mediators: their differential release and the secretory pathways involved. Front. Immunol..

[70] Stone KD, Prussin C, Metcalfe DD (2010). IgE, mast cells, basophils, and eosinophils. J. Allergy Clin. Immunol..

[71] Stingl G, Maurer D (1997). IgE-mediated allergen presentation via Fc epsilon RI on antigen-presenting cells. Int. Arch. Allergy Immunol..

[72] Selb R (2017). CD23 surface density on B cells is associated with IgE levels and determines IgE-facilitated allergen uptake, as well as activation of allergen-specific T cells. J. Allergy Clin. Immunol..

[73] Westman S, Gustavsson S, Heyman B (1997). Early expansion of secondary B cells after primary immunization with antigen complexed with IgE. Scand. J. Immunol..

[74] Gustavsson S, Hjulstrom S, Liu T, Heyman B (1994). CD23/IgE-mediated regulation of the specific antibody response in vivo. J. Immunol..

[75] Maezawa Y (2004). IgE-dependent enhancement of Th2 cell-mediated allergic inflammation in the airways. Clin. Exp. Immunol..

[76] Ceponis PJ, Botelho F, Richards CD, McKay DM (2000). Interleukins 4 and 13 increase intestinal epithelial permeability by a phosphatidylinositol 3-kinase pathway. Lack of evidence for STAT 6 involvement. J. Biol. Chem..

[77] Gutowska-Owsiak, D. et al. Histamine exerts multiple effects on expression of genes associated with epidermal barrier function. *J. Invest. Allergy Clin. Immunol*. **24**, 231–239 (2014). 25219105

[78] Gutowska-Owsiak D (2012). IL-17 downregulates filaggrin and affects keratinocyte expression of genes associated with cellular adhesion. Exp. Dermatol..

[79] Gutowska-Owsiak D, Schaupp AL, Salimi M, Taylor S, Ogg GS (2011). Interleukin-22 downregulates filaggrin expression and affects expression of profilaggrin processing enzymes. Br. J. Dermatol..

[80] Gutowska-Owsiak D, Ogg GS (2013). Cytokine regulation of the epidermal barrier. Clin. Exp. Allergy.

[81] Gschwandtner M (2013). Histamine suppresses epidermal keratinocyte differentiation and impairs skin barrier function in a human skin model. Allergy.

[82] Howell MD (2006). Mechanism of HBD-3 deficiency in atopic dermatitis. Clin. Immunol..

[83] Al-Sadi R, Boivin M, Ma T (2009). Mechanism of cytokine modulation of epithelial tight junction barrier. Front. Biosci..

[84] Leach L, Eaton BM, Westcott ED, Firth JA (1995). Effect of histamine on endothelial permeability and structure and adhesion molecules of the paracellular junctions of perfused human placental microvessels. Microvasc. Res..

[85] Walsh SV, Hopkins AM, Nusrat A (2000). Modulation of tight junction structure and function by cytokines. Adv. Drug Deliv. Rev..

[86] Ahdieh M, Vandenbos T, Youakim A (2001). Lung epithelial barrier function and wound healing are decreased by IL-4 and IL-13 and enhanced by IFN-gamma. Am. J. Physiol. Cell Physiol..

[87] Hirase T (2001). Regulation of tight junction permeability and occludin phosphorylation by Rhoa-p160ROCK-dependent and -independent mechanisms. J. Biol. Chem..

[88] Zabner J (2003). Histamine alters E-cadherin cell adhesion to increase human airway epithelial permeability. J. Appl. Physiol..

[89] Heller F (2005). Interleukin-13 is the key effector Th2 cytokine in ulcerative colitis that affects epithelial tight junctions, apoptosis, and cell restitution. Gastroenterology.

[90] Bogiatzi SI (2007). Cutting edge: proinflammatory and Th2 cytokines synergize to induce thymic stromal lymphopoietin production by human skin keratinocytes. J. Immunol..

[91] Kubo A, Nagao K, Amagai M (2012). Epidermal barrier dysfunction and cutaneous sensitization in atopic diseases. J. Clin. Invest..

[92] Albanesi C (2007). IL-4 and IL-13 negatively regulate TNF-alpha- and IFN-gamma-induced beta-defensin expression through STAT-6, suppressor of cytokine signaling (SOCS)-1, and SOCS-3. J. Immunol..

[93] Ong PY, Ohtake T, Brandt C (2002). Endogenous antimicrobial peptides and skin infections in atopic dermatitis. N. Engl. J. Med..

[94] Dabbagh K (1999). IL-4 induces mucin gene expression and goblet cell metaplasia in vitro and in vivo. J. Immunol..

[95] Atherton HC, Jones G, Danahay H (2003). IL-13-induced changes in the goblet cell density of human bronchial epithelial cell cultures: MAP kinase and phosphatidylinositol 3-kinase regulation. Am. J. Physiol. Lung Cell Mol. Physiol..

[96] Amrani Y, Chen H, Panettieri RA (2000). Activation of tumor necrosis factor receptor 1 in airway smooth muscle: a potential pathway that modulates bronchial hyper-responsiveness in asthma?. Respir. Res..

[97] Amrani Y, Krymskaya V, Maki C, Panettieri RA (1997). Mechanisms underlying TNF-alpha effects on agonist-mediated calcium homeostasis in human airway smooth muscle cells. Am. J. Physiol..

[98] Tliba O (2003). IL-13 enhances agonist-evoked calcium signals and contractile responses in airway smooth muscle. Br. J. Pharmacol..

[99] Nelson HS (2014). Subcutaneous immunotherapy versus sublingual immunotherapy: which is more effective?. J. Allergy Clin. Immunol. Pract..

[100] Aasbjerg K (2014). Immunological comparison of allergen immunotherapy tablet treatment and subcutaneous immunotherapy against grass allergy. Clin. Exp. Allergy.

[101] Schulten V (2016). Distinct modulation of allergic T cell responses by subcutaneous vs. sublingual allergen-specific immunotherapy. Clin. Exp. Allergy.

[102] Upadhyay J, Upadhyay RB, Agrawal P, Jaitley S, Shekhar R (2013). Langerhans cells and their role in oral mucosal diseases. N. Am. J. Med. Sci..

[103] Hovav AH (2014). Dendritic cells of the oral mucosa. Mucosal Immunol..

[104] Seneschal J (2012). Human epidermal langerhans cells maintain immune homeostasis in skin by activating skin resident regulatory T cells. Immunity.

[105] Till SJ (2001). Recruitment of CD1a+ Langerhans cells to the nasal mucosa in seasonal allergic rhinitis and effects of topical corticosteroid therapy. Allergy.

[106] Du Toit G (2013). Identifying infants at high risk of peanut allergy: the Learning Early about Peanut Allergy (LEAP) screening study. J. Allergy Clin. Immunol..

[107] Du Toit G (2015). Randomized trial of peanut consumption in infants at risk for peanut allergy. N. Engl. J. Med..

[108] Anagnostou K (2014). Assessing the efficacy of oral immunotherapy for the desensitisation of peanut allergy in children (STOP II): a phase 2 randomised controlled trial. Lancet.

[109] Cui ZH, Radinger M, Sjostrand M, Lotvall J (2012). Repeated allergen exposure reduce early phase airway response and leukotriene release despite upregulation of 5-lipoxygenase pathways. Clin. Transl. Allergy.

[110] Van Hove CL, Maes T, Joos GF, Tournoy KG (2007). Prolonged inhaled allergen exposure can induce persistent tolerance. Am. J. Respir. Cell Mol. Biol..

[111] Hourihane JOB, Roberts SA, Warner JO (1998). Resolution of peanut allergy: case-control study. Br. Med. J..

[112] Nowak-Węgrzyn A (2015). What makes children outgrow food allergy?. Clin. Exp. Allergy.

[113] Qamar N (2015). Naturally occurring tolerance acquisition to foods in previously allergic children is characterized by antigen specificity and associated with increased subsets of regulatory T cells. Clin. Exp. Allergy.

[114] Ponce M, Diesner SC, Szépfalusi Z, Eiwegger T (2016). Markers of tolerance development to food allergens. Allergy.

[115] Arts JH, Mommers C, de Heer C (2006). Dose-response relationships and threshold levels in skin and respiratory allergy. Crit. Rev. Toxicol..

[116] Arts JHE, Frieke Kuper C (2003). Approaches to induce and elicit respiratory allergy: impact of route and intensity of exposure. Toxicol. Lett..

[117] Friedmann PS (2007). The relationships between exposure dose and response in induction and elicitation of contact hypersensitivity in humans. Br. J. Dermatol..

[118] Robinson MK (2000). The importance of exposure estimation in the assessment of skin sensitization risk. Contact Dermatitis.

[119] Taylor SL (2010). Threshold dose for peanut: risk characterization based upon diagnostic oral challenge of a series of 286 peanut-allergic individuals. Food Chem. Toxicol..

[120] Chung YJ (2005). Dose-dependent allergic responses to an extract of Penicillium chrysogenum in BALB/c mice. Toxicology.

[121] Arts JHE, de Koning MW, Bloksma N, Kuper CF (2004). Respiratory allergy to trimellitic anhydride in rats: concentration-response relationships during elicitation. Inhal. Toxicol..

[122] Novak N (2012). Early suppression of basophil activation during allergen-specific immunotherapy by histamine receptor 2. J. Allergy Clin. Immunol..

[123] Sancho-Serra MdC, Simarro M, Castells M (2011). Rapid IgE desensitization is antigen specific and impairs early and late mast cell responses targeting FcεRI internalization. Eur. J. Immunol..

[124] Bin Dhuban K, Piccirillo CA (2015). The immunological and genetic basis of immune dysregulation, polyendocrinopathy, enteropathy, X-linked syndrome. Curr. Opin. Allergy Clin. Immunol..

[125] d’Hennezel E, Bin Dhuban K, Torgerson T, Piccirillo CA (2012). The immunogenetics of immune dysregulation, polyendocrinopathy, enteropathy, X linked (IPEX) syndrome. J. Med. Genet..

[126] Torgerson TR (2007). Severe food allergy as a variant of IPEX syndrome caused by a deletion in a noncoding region of the FOXP3 gene. Gastroenterology.

[127] Halabi-Tawil M (2009). Cutaneous manifestations of immune dysregulation, polyendocrinopathy, enteropathy, X-linked (IPEX) syndrome. Br. J. Dermatol..

[128] Collison LW (2010). Interleukin-35-mediated induction of a novel regulatory T cell population. Nat. Immunol..

[129] Kappen, J. H., Durham, S. R., Veen, H. I. & Shamji, M. H. Applications and mechanisms of immunotherapy in allergic rhinitis and asthma. *Ther. Adv. Respir. Dis*. **11**, 73–86 (2017). 10.1177/1753465816669662 PMC5941975 27678500

[130] Jutel M (2003). IL-10 and TGF-β cooperate in the regulatory T cell response to mucosal allergens in normal immunity and specific immunotherapy. Eur. J. Immunol..

[131] Fu C-L, Ye Y-L, Lee Y-L, Chiang B-L (2003). Both allergen-specific CD4 and CD8 Type 2 T cells decreased in asthmatic children with immunotherapy. Pediatr. Allergy Immunol..

[132] Caridade, M., Graca, L. & Ribeiro, R. Mechanisms underlying CD4+Treg immune regulation in the adult: from experiments to models. *Front. Immunol*. **4**, 378 (2013). 10.3389/fimmu.2013.00378 PMC3831161 24302924

[133] Saxena A (2015). Interleukin-10 paradox: a potent immunoregulatory cytokine that has been difficult to harness for immunotherapy. Cytokine.

[134] Ng, T. H. S. et al. Regulation of adaptive immunity; the role of interleukin-10. *Front. Immunol*. **4**, 129 (2013). 10.3389/fimmu.2013.00129 PMC3668291 23755052

[135] Varney VA (1993). Influence of grass pollen immunotherapy on cellular infiltration and cytokine mRNA expression during allergen-induced late-phase cutaneous responses. J. Clin. Invest..

[136] Bellinghausen I (1997). Insect venom immunotherapy induces interleukin-10 production and a Th2-to-Th1 shift, and changes surface marker expression in venom-allergic subjects. Eur. J. Immunol..

[137] McHugh SM, Deighton J, Stewart AG, Lachmann PJ, Ewan PW (1995). Bee venom immunotherapy induces a shift in cytokine responses from a TH-2 to a TH-1 dominant pattern: comparison of rush and conventional immunotherapy. Clin. Exp. Allergy.

[138] Jutel M (1995). Bee venom immunotherapy results in decrease of IL-4 and IL-5 and increase of IFN-gamma secretion in specific allergen-stimulated T cell cultures. J. Immunol..

[139] Maggi E (2010). T cell responses induced by allergen-specific immunotherapy. Clin. Exp. Immunol..

[140] Ryan JF (2016). Successful immunotherapy induces previously unidentified allergen-specific CD4+T-cell subsets. Proc. Natl. Acad. Sci. U. S. A..

[141] Gardner LM, O’Hehir RE, Rolland JM (2004). High dose allergen stimulation of T cells from house dust mite-allergic subjects induces expansion of IFN-gamma+T cells, apoptosis of CD4+IL-4+T cells and T cell anergy. Int. Arch. Allergy Immunol..

[142] Wambre E (2014). Specific immunotherapy modifies allergen-specific CD4(+) T-cell responses in an epitope-dependent manner. J. Allergy Clin. Immunol..

[143] Murthy PK, Dennis VA, Lasater BL, Philipp MT (2000). Interleukin-10 modulates proinflammatory cytokines in the human monocytic cell line THP-1 stimulated with Borrelia burgdorferi lipoproteins. Infect. Immun..

[144] Onishi Y, Fehervari Z, Yamaguchi T, Sakaguchi S (2008). Foxp3+ natural regulatory T cells preferentially form aggregates on dendritic cells in vitro and actively inhibit their maturation. Proc. Natl. Acad. Sci..

[145] Sojka DK, Hughson A, Sukiennicki TL, Fowell DJ (2005). Early kinetic window of target T cell susceptibility to CD25+ regulatory T cell activity. J. Immunol..

[146] Bopp T (2007). Cyclic adenosine monophosphate is a key component of regulatory T cell-mediated suppression. J. Exp. Med..

[147] Murakami Y (2013). Remarkable role of indoleamine 2,3-dioxygenase and tryptophan metabolites in infectious diseases: potential role in macrophage-mediated inflammatory diseases. Mediators Inflamm..

[148] Nakamura T (2007). Expression of indoleamine 2, 3-dioxygenase and the recruitment of Foxp3-expressing regulatory T cells in the development and progression of uterine cervical cancer. Cancer Sci..

[149] Sharma MD (2007). Plasmacytoid dendritic cells from mouse tumor-draining lymph nodes directly activate mature Tregs via indoleamine 2,3-dioxygenase. J. Clin. Invest..

[150] Bopp T (2007). Cyclic adenosine monophosphate is a key component of regulatory T cell-mediated suppression. J. Exp. Med..

[151] Klein M, Bopp T (2016). Cyclic AMP represents a crucial component of Treg cell-mediated immune regulation. Front. Immunol..

[152] Borsellino G (2007). Expression of ectonucleotidase CD39 by Foxp3+Treg cells: hydrolysis of extracellular ATP and immune suppression. Blood.

[153] Ring S, Oliver SJ, Cronstein BN, Enk AH, Mahnke K (2009). CD4+CD25+regulatory T cells suppress contact hypersensitivity reactions through a CD39, adenosine-dependent mechanism. J. Allergy Clin. Immunol..

[154] Grossman WJ (2004). Human T regulatory cells can use the perforin pathway to cause autologous target cell death. Immunity.

[155] Jones TG, Finkelman FD, Austen KF, Gurish MF (2010). T regulatory cells control antigen-induced recruitment of mast cell progenitors to the lungs of C57BL/6 mice. J. Immunol..

[156] Kwon DS (2012). CD4+CD25+regulatory T cells impair HIV-1-specific CD4 T cell responses by upregulating interleukin-10 production in monocytes. J. Virol..

[157] Taams LS (2005). Modulation of monocyte/macrophage function by human CD4+CD25+regulatory T cells. Hum. Immunol..

[158] Tiemessen MM (2007). CD4+CD25+Foxp3+regulatory T cells induce alternative activation of human monocytes/macrophages. Proc. Natl. Acad. Sci..

[159] Ganeshan K, Bryce PJ (2012). Regulatory T cells enhance mast cell production of IL-6 via surface-bound TGFβ(). J. Immunol..

[160] Gri G (2008). CD4+CD25+regulatory T cells suppress mast cell degranulation and allergic responses through OX40-OX40L interaction. Immunity.

[161] Baru AM (2010). Selective depletion of Foxp3+Treg during sensitization phase aggravates experimental allergic airway inflammation. Eur. J. Immunol..

[162] van de Veen W (2016). Role of regulatory B cells in immune tolerance to allergens and beyond. J. Allergy Clin. Immunol..

[163] Braza F, Chesne J, Castagnet S, Magnan A, Brouard S (2014). Regulatory functions of B cells in allergic diseases. Allergy.

[164] Boonpiyathad, T. et al. High-dose bee venom exposure induces similar tolerogenic B-cell responses in allergic patients and healthy beekeepers. *Allergy* **72**, 407–415 (2017). 10.1111/all.12966 27341567

[165] van de Veen W (2013). IgG4 production is confined to human IL-10-producing regulatory B cells that suppress antigen-specific immune responses. J. Allergy Clin. Immunol..

[166] Flicker S, Valenta R (2003). Renaissance of the blocking antibody concept in type I allergy. Int. Arch. Allergy Immunol..

[167] Munro-Ashman D, McEwen H, Feinberg JG (1971). The patient self (P-S) test. Demonstration of a rise in blocking antibodies after treatment with Allpyral. Int. Arch. Allergy Appl. Immunol..

[168] Cuthbert OD (1975). Application of the patient-self (P-S) test to assess the specificity of hyposensitization with Allpyral-G. Clin. Exp. Allergy.

[169] Blair H, Ezeoke A, Hobbs JR (1975). IgE, IgG and patient-self tests during slow hyposensitization to grass pollen. Clin. Exp. Allergy.

[170] Wilson DR (2001). Grass pollen immunotherapy inhibits seasonal increases in basophils and eosinophils in the nasal epithelium. Clin. Exp. Allergy.

[171] Ventura I (2014). Allergen immunotherapy decreases LPS-induced NF-kappaB activation in neutrophils from allergic patients. Pediatr. Allergy Immunol..

[172] Aroca, R. et al. Immunotherapy reduces allergen-mediated CD66b expression and myeloperoxidase levels on human neutrophils from allergic patients. *PLoS ONE*, **9** e94558 (2014). 10.1371/journal.pone.0094558 PMC3989194 24740105

[173] Passalacqua G (1995). Nasal immunotherapy to Parietaria: evidence of reduction of local allergic inflammation. Am. J. Respir. Crit. Care Med..

[174] Yu S-J, Liao E-C, Tsai J-J (2015). Effects of local nasal immunotherapy in allergic airway inflammation: using urea denatured Dermatophagoides pteronyssinus. Hum. Vaccin. Immunother..

[175] Lao-Araya M, Steveling E, Scadding GW, Durham SR, Shamji MH (2014). Seasonal increases in peripheral innate lymphoid type 2 cells are inhibited by subcutaneous grass pollen immunotherapy. J. Allergy Clin. Immunol..

[176] Hussey Freeland DM, Fan-Minogue H, Spergel JM, Chatila TA, Nadeau KC (2016). Advances in food allergy oral immunotherapy: toward tolerance. Curr. Opin. Immunol..

[177] Johansen P, von Moos S, Mohanan D, Kündig TM, Senti G (2012). New routes for allergen immunotherapy. Hum. Vaccin. Immunother..

[178] Senti G, von Moos S, Kundig TM (2014). Epicutaneous immunotherapy for aeroallergen and food allergy. Curr. Treat. Options Allergy.

[179] Hylander T, Latif L, Petersson-Westin U, Cardell LO (2013). Intralymphatic allergen-specific immunotherapy: an effective and safe alternative treatment route for pollen-induced allergic rhinitis. J. Allergy Clin. Immunol..

[180] Senti G, Johansen P, Kundig TM (2009). Intralymphatic immunotherapy. Curr. Opin. Allergy Clin. Immunol..

[181] Yesil, O. et al. Intranasal and intragastric allergen immunotherapy prevented chronic histopathologic changes in a murine model of asthma. *J. Allergy Clin. Immunol*. **113**, S210 (2004).

[182] Durham SR (2012). SQ-standardized sublingual grass immunotherapy: confirmation of disease modification 2 years after 3 years of treatment in a randomized trial. J. Allergy Clin. Immunol..

[183] Reich K (2011). Immunologic effects and tolerability profile of in-season initiation of a standardized-quality grass allergy immunotherapy tablet: a phase III, multicenter, randomized, double-blind, placebo-controlled trial in adults with grass pollen–induced rhinoconjunctivitis. Clin. Ther..

[184] Valenta R, Campana R, Focke-Tejkl M, Niederberger V (2016). Vaccine development for allergen-specific immunotherapy based on recombinant allergens and synthetic allergen peptides: lessons from the past and novel mechanisms of action for the future. J. Allergy Clin. Immunol..

[185] Spertini F (2014). Safety and immunogenicity of immunotherapy with Bet v 1-derived contiguous overlapping peptides. J. Allergy Clin. Immunol..

[186] Spertini F (2016). Efficacy of 2 months of allergen-specific immunotherapy with Bet v 1-derived contiguous overlapping peptides in patients with allergic rhinoconjunctivitis: results of a phase IIb study. J. Allergy Clin. Immunol..

[187] Pellaton C (2013). Novel birch pollen specific immunotherapy formulation based on contiguous overlapping peptides. Clin. Transl. Allergy.

[188] Creticos PS (2014). Advances in synthetic peptide immuno-regulatory epitopes. World Allergy Organ. J..

[189] Marth K (2013). A nonallergenic birch pollen allergy vaccine consisting of hepatitis PreS-fused Bet v 1 peptides focuses blocking IgG toward IgE epitopes and shifts immune responses to a tolerogenic and Th1 phenotype. J. Immunol..

[190] Valenta R, Campana R, Focke-Tejkl M, Niederberger V (2016). Vaccine development for allergen-specific immunotherapy based on recombinant allergens and synthetic allergen peptides: lessons from the past and novel mechanisms of action for the future. J. Allergy Clin. Immunol..

[191] Maasch HJ, Marsh DG (1987). Standardized extracts modified allergens—allergoids. Clin. Rev. Allergy.

[192] Niederberger V (2004). Vaccination with genetically engineered allergens prevents progression of allergic disease. Proc. Natl. Acad. Sci. U. S. A..

[193] Jutel M (2013). Mechanisms of allergen-specific immunotherapy and novel ways for vaccine development. Allergol. Int..

[194] Focke-Tejkl, M. et al. Development and characterization of a recombinant, hypoallergenic, peptide-based vaccine for grass pollen allergy. *J. Allergy Clin. Immunol*. **135**, 1207-1217.e1211 (2015). 10.1016/j.jaci.2014.09.012 PMC4418753 25441634

[195] Massanari M (2010). Effect of pretreatment with omalizumab on the tolerability of specific immunotherapy in allergic asthma. J. Allergy Clin. Immunol..

[196] Shi Y (2015). Specific immunotherapy in combination with Clostridium butyricum inhibits allergic inflammation in the mouse intestine. Sci. Rep..

